# Simulation Analysis and Experimental Verification of High-Speed Impact of Rocky Asteroids

**DOI:** 10.3390/s25072055

**Published:** 2025-03-25

**Authors:** Fan Huang, Zhiqing Geng, Binqiang Luo, Yuming Peng, Liang Xu, Wei Wang, Biyue Pan, Dongyu Li

**Affiliations:** 1 School of Instrument science and Engineering, Southeast University, Nanjing 201100, China; huangfan1990.ok@163.com; 2 Shanghai Institute of Satellite Engineering, Shanghai 201100, China; fdyzgzq@126.com (Z.G.); pym328311@126.com (Y.P.); hmxl105@126.com (L.X.); deepspace509@126.com (W.W.); dongyuli@buaa.edu.cn (D.L.); 3 School of Mechanical Engineering, Shanghai Jiao Tong University, Shanghai 201100, China; 4 Institute of Fluid Physics, China Academy of Engineering Physics, Mianyang 621000, China; bqluoo@caep.cn; 5 School of Cyber Science and Technology, Beihang University, Beijing 102206, China

**Keywords:** asteroid defense, kinetic impact, momentum transfer coefficient, SPH, impact simulation

## Abstract

Kinetic impact is an effective way to deal with threatening asteroids, and the momentum transfer coefficient during the impact process is an effective indicator for evaluating the impact effect. This article is based on the use of the Smoothed Particle Hydrodynamics (SPH) method to establish a simulation model of high-speed impact of flying discs on granite targets, and obtain parameters such as the shape of the splashing material and the distribution of the target damage during the impact process. An analysis was conducted on the influence of different impact velocities on the kinetic energy transfer coefficient, and it was found that the momentum transfer coefficient increased with the increase in impact velocity, from 1.59 at 5 km/s to 1.96 at 11.7 km/s. A ground high-speed impact system with a speed of over 10 km/s has been established, and the actual momentum transfer coefficient has increased from 1.73 at 7 km/s to around 2.06 at 11.7 km/s. The variation trend of kinetic energy transfer coefficients obtained from experiments and simulations is consistent, with an error of basically within 10%, and the simulation results are effective. The simulation and experimental analysis of high-speed kinetic impact can provide a reference for the engineering implementation of asteroid impact defense missions.

## 1. Introduction

There are a large number of Potentially Hazardous Asteroids (PHAs) near Earth, posing a serious threat to the safety of the planet and human survival. At present, various asteroid defense methods have been proposed, but most of them are still in the early conceptual research stage [1]. Due to technological or other limitations, these methods may be difficult to transform into practical scientific tasks for verification in a short period of time. Directly launching impactors for impact is currently the easiest and most mature method to implement [2]. Kinetic impact refers to the high-speed impact of a spacecraft on an asteroid at a certain angle, causing the asteroid’s orbit to deviate and move away from Earth [3]. From the aspects of technological maturity and operability, it is considered the simplest and most feasible disposal technology currently available. On 26 September 2022, NASA’s Double Asteroid Redirection Test (DART) probe successfully impacted the target asteroid Dimorphos, causing it to deviate from its original orbit. This was the world’s first successful planetary defense test mission, verifying the feasibility of a kinetic impact technology on planets [4,5,6].

The complex and drastic changes during the high-speed impact of asteroids are difficult to solve directly through analysis, and are mainly studied through numerical simulations and experiments [7]. Ground experiments are used to verify the effectiveness of simulation results, and to predict experimental results through simulation, guiding the formulation of experimental parameters. At present, numerical simulation methods for high-speed collision dynamics can be divided into gridded methods and meshless methods according to gridding standards [8]. The gridding method replaces the continuous solution region with a grid composed of a finite number of discrete points, and then uses the difference quotient of grid nodes instead of the derivative, directly transforming the differential equation into an algebraic equation for solution, such as the Lagrangian method and Euler method [9,10]. Although grid based numerical methods have achieved great success, they are difficult to use to handle discontinuous problems such as large deformations, motion interfaces, and free surfaces [11].

The meshless method does not use a pre-defined grid structure to discretize the solution domain, but instead replaces the grid structure with a series of node arrangements [12]. An approximation related to the weight function is used to represent the physical information on the nodes. A node on the solution domain can affect the mechanical properties of any point on the entire solution domain, thus breaking away from the concept of elements or grids in numerical calculations. Among them, SPH is a classic meshless method that can handle problems with large deformations, deformation boundaries, free surfaces, and motion interfaces [13,14,15]. It has been increasingly widely used in high-speed impact dynamics simulations.

At present, the international technology for achieving ultra-high-speed launches exceeding 10 km/s is very limited, mainly including three-stage light gas guns, electric guns, magnetic driven flyers, and directional energy gathering acceleration technology [16]. Among them, electric guns are loading devices that use high-pressure gas generated by metal foil electric explosions to drive the ultra-high-speed movement of plastic flyers. Lawrence Livermore National Laboratory (LLNL) in the United States conducted in-depth research on electric guns, which led to the development of the diameter of the fins from a few millimeters to hundreds of millimeters, and achieved ultra-high-speed firing of approximately 43 mg fins at 18 km/s on its 100 kV electric gun device [17]. The Fluid Physics Research Institute of China Academy of Engineering Physics has successively established electric cannon devices that can adapt to various conditions and conducted experimental research on metal foil electric explosion-driven ultra-high-speed flying discs. Its 98 kJ electric cannon device and 200 kJ electric cannon device, respectively, drive 22 mg and 242 mg flying discs to 10 km/s, which can be used for ground high-speed impact test research [18].

This article first establishes a simulation model of high-speed impact based on the SPH method, and obtains parameters such as the shape of the debris cloud and the distribution of the asteroid damage during the impact process; secondly, forward collision simulations were conducted at different speeds, and the influence of impact velocity on momentum transfer coefficient was obtained; we established a high-speed impact experimental system, conducted ground high-speed impact experiments, and compared and analyzed the simulation results to verify the effectiveness of the simulation results, which can provide useful references for the implementation of engineering tasks.

## 2. Impact Process and Efficiency Evaluation

**Figure 1:**
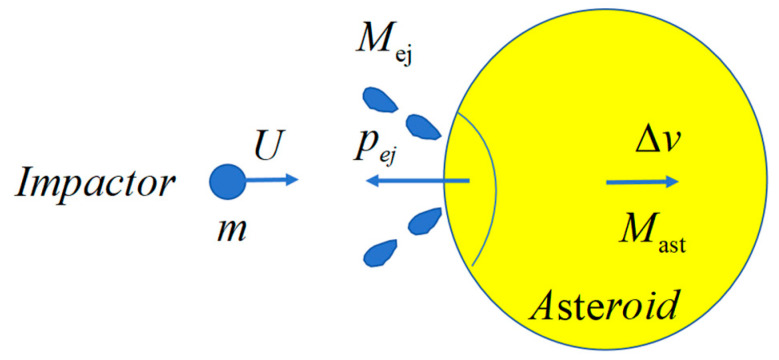
Principle of an impact deflection.

As shown in Figure 1, the process of orbit disposal using impact can be divided into three stages: (1) the transfer of the impactor before the collision, (2) the disposal operations during the impact, and (3) the orbital deflection of the small celestial body after the impact. During the process of colliding with the target small celestial body, the impactor will transfer momentum with the small celestial body. At the same time, under high-speed impact, the material on the surface of the target small celestial body will be broken, producing impact ejecta, further enhancing the momentum transfer effect generated by the impact.

The conservation of momentum during the impact process can be expressed as follows:

(1) $$mU+p_{\mathrm{ej}}\approx M_{\mathrm{ast}}\Delta v$$

Among them, $M_{\mathrm{ast}}$ is the target mass, $p_{\mathrm{ej}}$ is the momentum of the anti splash in the opposite direction of the projectile’s incidence, and Δv is the change in target velocity. Usually, the mass of the target is much greater than that of the projectile and splash, so the change in target mass before and after impact can be ignored. It can be seen that in order to obtain the change in target velocity after impact, in addition to direct measurement Δv methods, the key is to determine the momentum of the anti splash $p_{\mathrm{ej}}$ or momentum transfer coefficient:

(2) $$\beta =\frac{M_{\mathrm{ast}}\Delta v}{mU}=1+\frac{p_{\mathrm{ej}}}{mU}$$

The larger the momentum transfer coefficient, the greater the momentum obtained by the asteroid, and the impact effect is about significant.

## 3. SPH Numerical Simulation Method

The SPH method is a numerical approach for solving partial differential equations and is a type of meshless method. This numerical method first discretizes the solution domain of the partial differential equation, then uses an approximate function to represent the field function and its derivative at any point, thereby transforming the partial differential equation into a series of discretized, time-dependent ordinary differential equations. Finally, these ordinary differential equations are solved using traditional numerical methods to obtain the numerical solution of the problem [9,19,20].

### 3.1. Smooth Particle Hydrodynamics Approximation

The governing equations of continuum mechanics under no external force using Lagrangian descriptions include the following series of equations.

Conservation of mass equation:

(3) $$\frac{d\rho }{dt}=-\rho \frac{𝜕v^{\beta }}{𝜕x^{\beta }}$$

Momentum conservation equation:

(4) $$\frac{dv^{\alpha }}{dt}=\frac{1}{\rho }\frac{𝜕\sigma^{\alpha \beta }}{𝜕x^{\beta }}$$

Energy conservation equation:

(5) $$\frac{de}{\;dt}=\frac{\sigma^{\alpha \beta }}{\rho }\frac{𝜕v^{\alpha }}{𝜕x^{\beta }}$$

Here, x denotes the spatial position vector, v represents the velocity vector, σ is the total stress tensor, and superscripts α and β indicate the spatial coordinate directions. ρ stands for density, e represents internal energy, and t denotes time. The SPH method is applied to discretize and approximate the fundamental conservation equations of continuum mechanics in the spatial domain.

The discrete form of the conservation of mass equation is as follows:

(6) $$\frac{d\rho_{i}}{\;dt}=-\rho_{i}\sum_{j=1}^{N}\frac{m_{j}}{\rho_{j}}v_{j}^{\beta }\cdot \frac{𝜕W_{ij}}{𝜕x_{i}^{\beta }}$$

The discrete form of the momentum conservation equation is as follows:

(7) $$\frac{dv_{i}^{\alpha }}{dt}=\frac{1}{\rho_{i}}\sum_{j=1}^{N}\frac{m_{j}}{\rho_{j}}\sigma_{j}^{\alpha \beta }\cdot \frac{𝜕W_{ij}}{𝜕x_{i}^{\beta }}$$

The discrete form of the energy conservation equation is as follows:

(8) $$\frac{de_{i}}{\;dt}=\frac{\sigma_{i}^{\alpha \beta }}{\rho_{i}}\sum_{j=1}^{N}\frac{m_{j}}{\rho_{j}}v_{j}^{\alpha }\cdot \frac{𝜕W_{ij}}{𝜕x_{i}^{\beta }}$$

Here, i and j represent the i-th and j-th particles, N is the total number of particles in the tightly supported domain of particle i, and $W_{ij}$ represents the smooth function of particle j affecting particle i.

### 3.2. Artificial Viscosity and Equation of State

Artificial viscosity is commonly used in shock wave calculations to maintain the stability of SPH solutions. Currently, the most widely used is the Monaghan type artificial viscosity in SPH method, which not only converts kinetic energy into thermal energy, providing essential dissipation for shock wave surfaces, but also prevents non-physical penetration when particles approach each other.

The equation of state (EOS) is a formula that characterizes the relationship between pressure, density, and temperature within a fluid. During high-speed impact, the shear effect of materials under high pressure can be ignored, and solids will exhibit fluid properties. The response of materials can be described by thermodynamic parameters. Commonly used EOSs include linear polynomials, JWL(Jones–Wilkins–Lee), Gruneisen, etc. In addition, some material models, such as the Johnson–Holmquist Concrete (HJC) model, include material strength models, damage models, and EOS, where the EOS includes three stages: elastic compression, compaction deformation, and post compaction deformation.

## 4. Numerical Simulation of Impact Process

### 4.1. Simulation Process

The process of asteroid impact involves transient, large deformation, large strain, material failure, and even complete failure or complex contact structural problems [21]. LS-DYNA has powerful analysis functions and rich material models, and has developed into one of the most famous universal multi physics field dynamics analysis software in the world, which can quickly solve multiple nonlinear contact collision problems.

When using the LS-DYNA solver for calculations, it is necessary to use both pre-processing software and post-processing software simultaneously. Commercial software such as ANSYS/ABAQUS or LS-DYNA’s built-in LS Prepost software can be used to define the modeling, meshing, and contact relationships of the model. After converting them into keyword files, the model materials, solution process control, output file format, and other key parameters can be set in LS Prepost (Version 4.3). Finally, the complete keyword file is submitted to the LS-DYNA solver (Version R11) for calculation. The result file is input into the post-processing software LS Prepost for analysis of stress, strain, velocity, overload, and displacement curves.

### 4.2. Simulation Model

**Figure 2:**
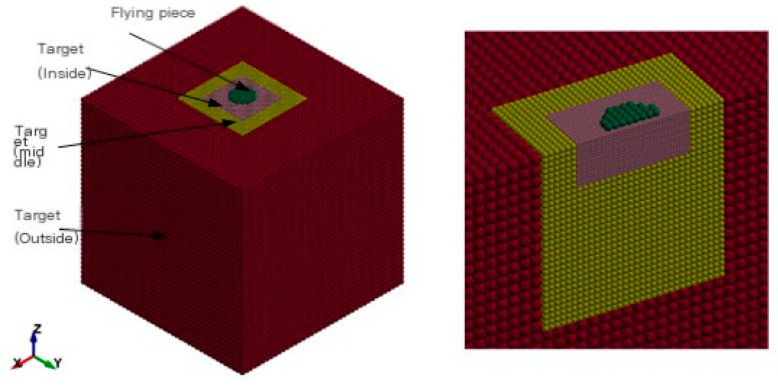
SPH model for impact simulation.

The LS-DYNA finite element simulation software was used to establish a model of the flying disc impacting the target, as shown on the left side of Figure 2. The flying disc is a polyester film cylinder with a size of 7 φ 12 × 0.3 mm, a density of 1.2 g/cm^3^, and a total weight of approximately 0.041 g; the target is cubic granite with a size of 10 × 10 × 10 cm, a density of 2.85 g/cm^3^, and a total mass of 2850 g. Both the flying disc and the target are simulated using SPH particles. To ensure calculation accuracy, the contact area between the flying disc and the target should maintain a similar particle density. At the same time, to reduce the computational workload, the particle density should not be too high. Therefore, the target is set to three layers, as shown on the right side of Figure 2. The particles in the central area are the most dense, and they become sparser as they move outward. The number of target particles is 115,072, the number of flying particles is 52, and the total number of particles is 115,124.

The parameters in the LS-DYNA solver have no units. Users can unify the parameter units with the model size units based on the solving model. On the one hand, the impact penetration time of the seven hit device is extremely short, requiring only milliseconds to complete; on the other hand, choosing a smaller series time unit can indicate a larger 3-complex value, which is beneficial for step-by-step integration. Therefore, cm−g−μs was chosen as the basic unit in the simulation.

### 4.3. Material Selection

In high-speed impact problems, it is necessary to consider the elastic–plastic relationship of materials under dynamic loads. Commonly used constitutive models for elastoplastic materials include ideal elastoplastic, bilinear elastoplastic, exponential hardening elastoplastic, and multilinear elastoplastic. Among them, the index hardening elastoplastic model (POWER_LAW-PLASTICITY) is a commonly used material constitutive model for plastic materials, which generates plastic hardening according to exponential observations after the material reaches its yield limit. The impactor adopts an exponential hardening elastoplastic constitutive model, which simulates the structural response of materials under high impact overload by setting strengthening coefficients, hardening indices, etc., as shown in Table 1 [22].

The Johnson–Holmquist Concrete (HJC) model was proposed in 1993 based on the Johnson–Cook model to describe the constitutive behavior and parameters of rock and soil [23]. The equivalent strength of rock and soil is expressed as a function of pressure, strain rate, and damage, where pressure is expressed as a function of volumetric strain, and the effect of permanent crushing is considered. The HJC model considers the effects of strain rate, hydrostatic pressure, and damage accumulation on strength and is widely used in numerical calculations. Using HJC’s built-in failure criteria, FS < 0 is set to select the degree of damage, D, to control failure. On the other hand, due to the inadequacy of the HJC constitutive model in the static water zone, the minimum pressure at failure (MNPRES) = −7.4 × 10^−5^ Mbar, the maximum principal strain at failure (MXEPS) = 0.35, and the maximum shear strain at failure (EPSSH) = 0.28 are added. The granite target material adopts the material parameters in Table 2 [22].

### 4.4. Impact Process

**Figure 3:**
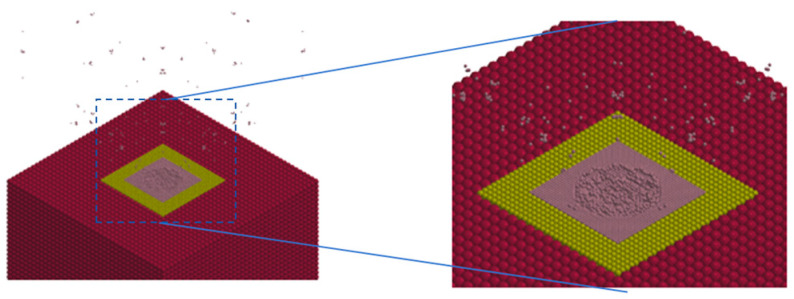
Shape of splashes during the impact process.

Consider the process of a flying disc colliding head on. At an impact velocity of 7 km/s, the impact process is shown in Figure 3. Within a few milliseconds, the flying disc completes its contact and destruction with the asteroid, and the flying disc and target fragments are ejected outward. The target gains momentum from the flying disc and debris. Due to the small mass of the flying disc, it only creates shallow impact craters on the target surface.

**Figure 4:**
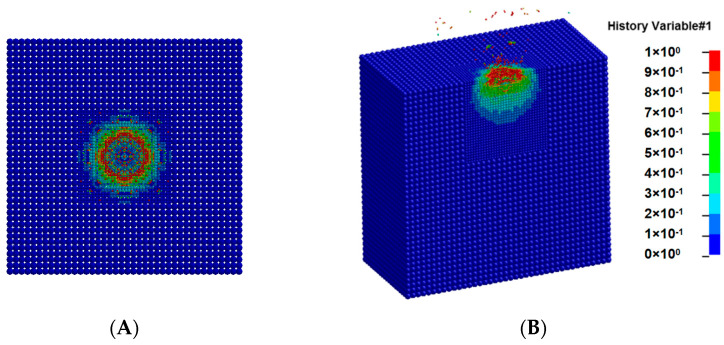
Shape of splashes during the impact process. (**A**) Top view. (**B**) Sectional view.

The damage distribution of the target during the impact process is shown in Figure 4. After the flying disc hits the granite, shock waves appear inside the granite and damage occurs. Due to the small thickness of the flying disc, the backward shock wave inside the flying disc quickly reaches the rear surface of the flying disc after impact, resulting in unloading. After the unloading wave is transmitted to the granite target, the damaged material is ejected backwards. As time goes by, the damage and destruction inside the granite gradually stop, and the anti spray material gradually separates from the granite target. When the impact speed increases, the overall impact process is similar, but the intensity increases. Due to the frontal collision, it can be seen from the top view, as shown in Figure 4A, that the damage generated by the target is a symmetrical circle. From the cross-sectional view, as shown in Figure 4B, it can be seen that the damage generated inside the target is approximately semi-spherical.

### 4.5. Effects of Different Impact Speeds

**Figure 5:**
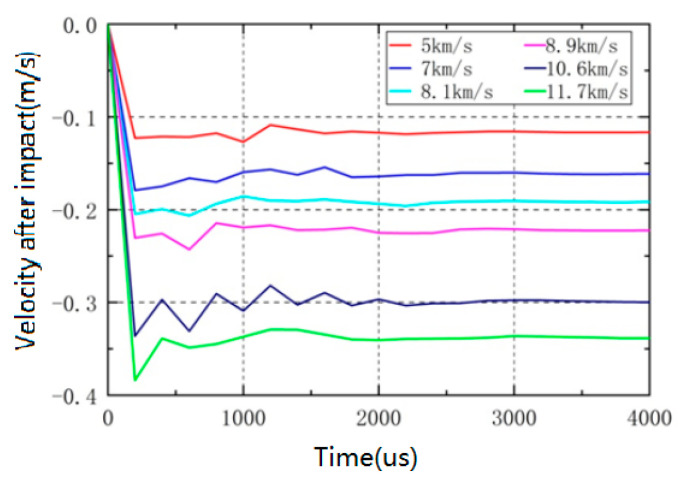
Velocity curves of targets at different impact velocities.

In order to compare with the actual impact test, simulation analysis was conducted on the working conditions at different impact velocities such as 5 km/s, 7 km/s, 8.1 km/s, 8.9 km/s, 10.6 km/s, and 11.7 km/s. The velocity curve of the granite target is shown in Figure 5. At different impact velocities, the movement trend of the target is basically consistent, reaching its maximum velocity at around 200 μs, followed by slight oscillations. After the impact ends, the elastic wave reflection inside the target gradually dissipates 1 ms later, and the granite target moves at the same velocity as a whole. The final velocity of the target is positively correlated with the impact velocity. When the impact velocity is 5 km/s, the target velocity is 0.117 m/s, and when the impact velocity is 11.7 km/s, the target velocity is 0.338 m/s.

Based on the velocity of the target after impact, the momentum obtained by the target can be calculated, divided by the momentum of the flying disc, to obtain the momentum transfer coefficient during the impact process. The momentum transfer coefficient increases with the increase in impact velocity, from 1.59 at 5 km/s to 1.96 at 11.7 km/s.

## 5. High-Speed Impact Ground Test

### 5.1. Experimental System Design

**Figure 6:**
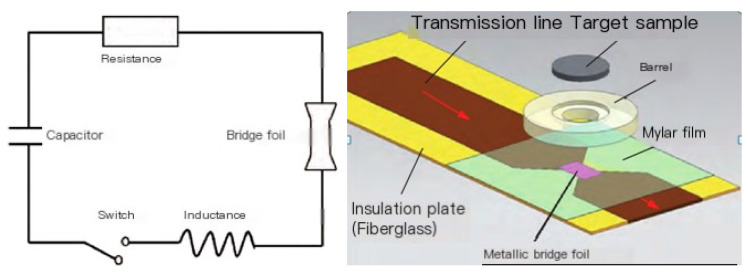
Schematic diagram of electric gun loading.

For ultra-high-speed launches over 7 km, electric cannon driven ultra-high-speed flyers are a mature launch technology, as shown in Figure 6. After the energy storage capacitor is charged, the external circuit instantly conducts and generates a pulsed high current in the discharge circuit. Under the action of a pulsed high current, the metal foil on the bridge foil plate is rapidly heated and undergoes melting gasification, ultimately leading to an electric explosion and the formation of high-temperature and high-pressure gas/plasma. The plastic sheet is cut into a circular flying disc by the acceleration chamber, and moves along the acceleration chamber bore under the drive of high-pressure gas and achieves high speed. At present, the Fluid Physics Research Institute of the Chinese Academy of Engineering Physics has established 98 kJ and 200 kJ electric cannon devices, with the ability to launch from Yak level flying disc to 10 km/s.

**Figure 7:**
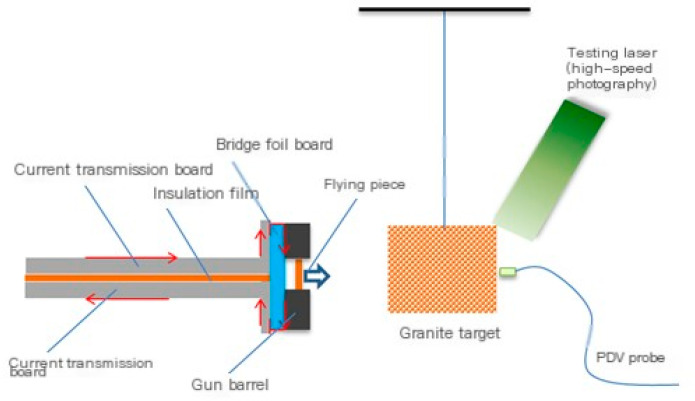
Experimental system diagram of metal foil electric explosion-driven flying disc impacting rock and soil targets.

The equivalent test verification system for high-speed kinetic impact on the ground is shown in Figure 7. Using a 98 kJ electric cannon device (Chinese Academy of Engineering Physics, Mianyang, China) to drive the flying disc, the target is suspended by high-strength soft ropes. Install two vertical stainless steel plates at the end of the current transmission plate, and connect the right end of the stainless steel plate to the copper transmission lines at the upper and lower ends of the bridge foil plate. The right surface of the bridge foil plate is covered with copper transmission lines and explosive metal foil. The metal foil is electrically exploded to drive the flying disc to fly to the right and collide with the suspended rock and soil target. High-speed photography is used to measure the impact velocity of the flying disc and the process of target fragmentation. High-Performance Tracking and Visualization (HPTV) is used to measure the distribution of splashing generated by the impact, and Photonic Doppler Velocimetry (PDV) is used to measure the velocity of the target after impact. By adjusting the charging voltage of the electric cannon device, the speed of the flying disc can be regulated.

**Figure 8:**
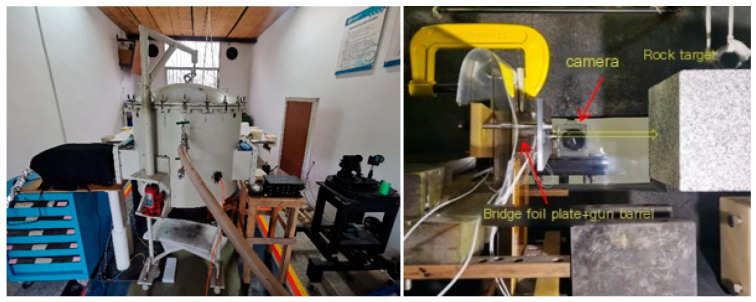
Experimental system and target room photos of electric cannon device.

When measuring the momentum transfer coefficient of ultra-high-speed flying discs impacting rock and soil targets, it is necessary to achieve high-speed flying discs in the horizontal direction to facilitate the velocity measurement of the target after impact. Therefore, the load structure of the 98 kJ electric cannon device was modified by changing the original horizontal installation of the bridge foil plates and vertical launch of the flying disc to a vertical installation of the bridge foil plates and forward launch of the flying disc. The experimental structure of the high-speed flying disc impact target driven by a 98 kJ electric cannon is shown in Figure 8.

### 5.2. Test Results

**Figure 9:**
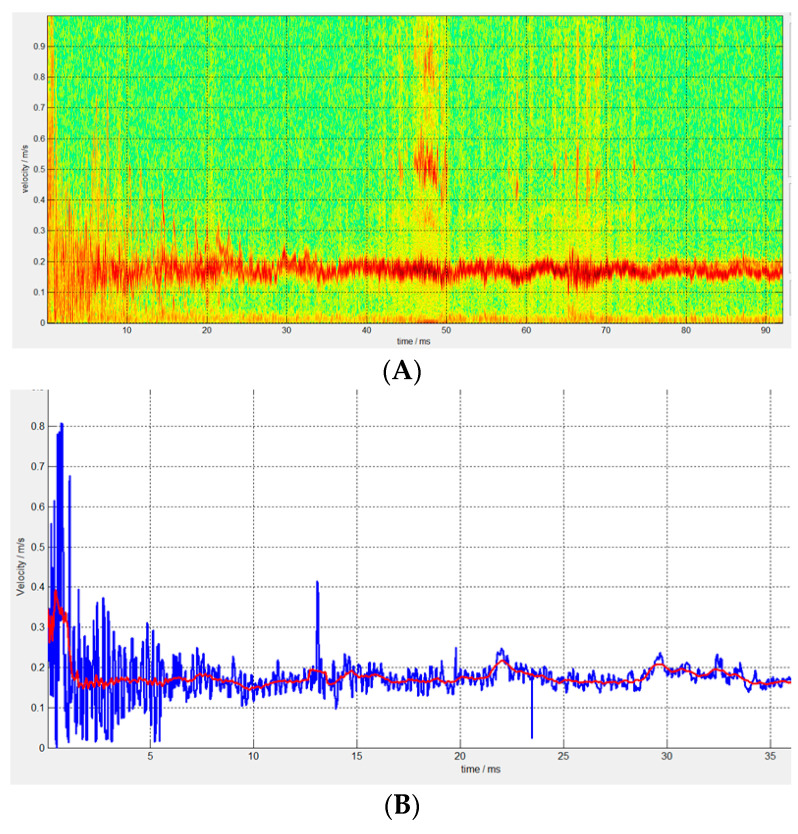
Velocity of granite target after impact (12 × 0.3 Flying Disc, 7 km/s). (**A**) PDV velocity spectrum. (**B**) The processed velocity curve.

To obtain momentum transfer coefficients at different impact velocities, ultra-high-speed flying discs were used to impact granite. The density of the granite material is 2.85 g/cm^3^, and the test target size is 10 cm × 10 cm × 10 cm. Figure 9 shows the velocity change in the granite target after being impacted by a φ 12 × 0.3 mm flying disc at 7 km/s. Figure 9A shows the velocity distribution of the target after impact measured using PDV technology. Figure 9B shows the velocity curve after signal extraction. After smoothing the data, the red curve represents the final velocity change curve of the target. Within 1 ms after the impact, the velocity of the granite target showed a significant decrease, and then remained stable for a long time. Compared with the simulation results in Figure 5, the velocity curve of the target after impact is basically consistent. The velocity changes rapidly at the moment of impact and eventually stabilizes at around 0.16 m/s. Due to the slight deviation between the simulation model and the actual weight of the target, there is a slight difference in the momentum transfer coefficient, but it is basically consistent.

The experimental results of the momentum transfer coefficient of a granite target under high-speed impact are shown in Table 3. The actual momentum transfer coefficient is basically positively correlated with the impact velocity, increasing from 1.73 at 7 km/s to around 2.06 at 11.7 km/s, indicating that the splashing generated by the impact increases the momentum of the target, and the impact effect is significant with the increase in impact velocity.

### 5.3. Comparative Analysis with Simulation Results

**Figure 10:**
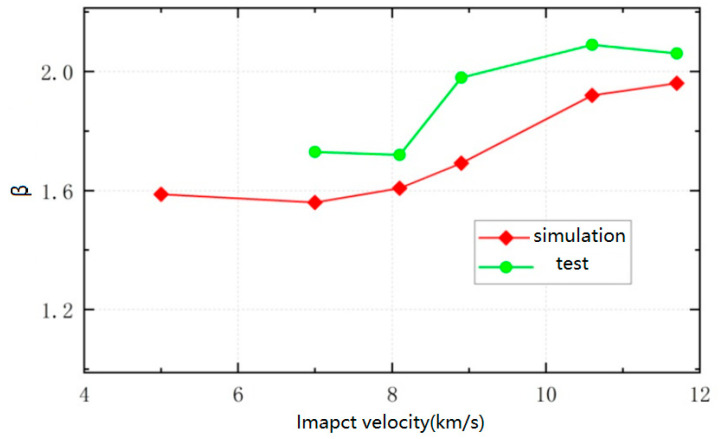
Momentum transfer coefficient of granite at different impact velocities.

The momentum transfer coefficients of polyester flyers hitting granite at different speeds obtained from experiments and simulations are shown in Figure 10. As can be seen from the figure, the trend of the experiment and simulation is basically consistent, and the momentum transfer coefficient increases with the increase in impact velocity. The values obtained from the simulation are relatively small compared to the experiment, with an error of 14.5% at 8.9 km/s and an error of within 10% for other operating conditions. The simulation analysis results are basically consistent with the experiment. The main reason for the existence of errors lies in the selection of material parameters, and there is a certain difference between the simulation parameters of polyester flyers and granite targets and the real parameters; at the same time, precise measurements of the initial velocity of polyester flyers and the velocity after impact with granite targets need to be carried out in the experiment, and there may be certain errors in the case of ultra-high speeds. Subsequently, simulation parameters need to be calibrated based on the measured material parameters to further reduce the error between the simulation and actual measurement, better simulate real impact situations through simulation, and provide a reference for the implementation of actual engineering tasks.

## 6. Conclusions

This article simulates the process of a flying disc impacting a granite target using LS-DYNA. The simulation shows that within a few milliseconds, the flying disc completes contact and destruction with the target, and the flying disc and target fragments are ejected outward. The target obtains momentum from the flying disc and projectile, which is consistent with the theoretical analysis results. The momentum transfer coefficient increases with the increase in impact velocity, from 1.59 at 5 km/s to 1.96 at 11.7 km/s. A ground high-speed impact system with a speed of over 10 km/s has been established, and the actual momentum transfer coefficient has increased from 1.73 at 7 km/s to around 2.06 at 11.7 km/s. The variation trend of the kinetic energy transfer coefficient obtained from experiments and simulations is consistent. Except for an error of 14.5% at 8.9 km/s, the error of other operating conditions is within 10%. The simulation process is effective and can basically reflect the actual impact process. Subsequently, the simulation parameters can be corrected based on the measured material parameters to further reduce simulation errors and provide a reference for the implementation of actual asteroid impact defense missions.

Although this study has achieved certain results in the kinetic energy simulation of asteroids, there is still room for improvement in the asteroid material and simulation condition design. In the future, we will further expand the types of asteroid materials and simulate the impact processes of more kinds of rocky and metallic asteroids to reveal the differences in their responses during impacts. Meanwhile, we will conduct refined simulations for various impact angles to analyze how angle variations affect energy transfer and destruction effects during impacts. Moreover, the optimization of impactor shapes will be a key focus of future research. By simulating different impactor shapes, we will explore their potential to enhance the deflection efficiency and impact effects on asteroids.

## Figures and Tables

**Table 1:** Parameters of the constitutive model for polyester film flyers.

Density ρ (g/cm^3^)	E/Mbar Elastic Modulus	Poisson’s Ratio *υ*	Enhancement Coefficient K	Hardening Index N	Plastic Failure Strain EPSF
1.2	0.0235	0.38	0.0147	0.192	3.0157

**Table 2:** Parameters of constitutive model for granite target body.

Parameters	Values	Parameters	Values	Parameters	Values
ρ (g/cm^3^)	T2.62	*G/*Mbar	0.23	*A*	0.17
*B*	2.4	*C*	0.005	*N*	0.75
*fc/*Mbar	1.08 × 10^−3^	*T/*Mbar	7.4 × 10^−5^	${\dot{\varepsilon }}_{0}/\mu s^{-1}$	1 × 10^−6^
*EFMIN*	0.01	*S*_MAX_	12	*P*_crush_ **/** *Mbar*	3.6 × 10^−4^
*μ*_crush_	0.001	*P*_lock_ **/** *Mbar*	0.015	*μ*_lock_	0.02
*D*_1_	0.04	*D*_2_	1	*K*_1_ **/** *Mbar*	0.79
*K*_2_ **/** *Mbar*	−3.04	*K*_3_ **/** *Mbar*	46.97	*FS*	−1

**Table 3:** Test for momentum transfer coefficient of targets under ultra-high-speed impact.

Serial Number	Bridge Foil/Flyer Size (mm)	Bore Size (mm)	Flying Speed (km/s)	Target Size (cm) and Mass (g)	Target Speed (m/s)	Momentum Transfer Coefficient
1	12 × 12 × 0.05/0.3	Φ 12 × 10	7.0	10 × 10 × 10, 3084	0.16	1.72
2	12 × 12 × 0.05/0.3	Φ 12 × 12	8.1	10 × 10 × 10, 2996	0.19	1.72
3	12 × 12 × 0.05/0.3	Φ 12 × 12	8.9	10 × 10 × 10, 2989	0.24	1.98
4	10 × 10 × 0.05/0.3	Φ 10 × 12	10.6	10 × 10 × 10, 2992	0.21	2.09
5	10 × 10 × 0.05/0.25	Φ 10 × 12	11.7	10 × 10 × 10, 2988	0.19	2.06

## Data Availability

Data is contained within the article.

## References

[1] Lubin P., Cohen A.N. (2023). Asteroid interception and disruption for terminal planetary defense. Adv. Space Res..

[2] Vasile M., Thiry N. (2016). Methods and Techniques for Asteroid Deflection. Asteroid and Space Debris Manipulation: Advances from the Stardust Research Network.

[3] Johnson L., Handal J., Fast K. (2023). A smashing success for planetary defence. Nat. Astron..

[4] Cheng A.F., Agrusa H.F., Barbee B.W., Meyer A.J., Farnham T.L., Raducan S.D., Richardson D.C., Dotto E., Zinzi A., Della Corte V. (2023). Momentum transfer from the DART mission kinetic impact on asteroid Dimorphos. Nature.

[5] Richardson D.C., Agrusa H.F., Barbee B., Cueva R.H., Ferrari F., Jacobson S.A., Makadia R., Meyer A.J., Michel P., Nakano R. (2024). The Dynamical State of the Didymos System Before and After the DART Impact.

[6] Nakano R., Hirabayashi M., Raducan S.D., Pravec P., Naidu S.P., Agrusa H.F., Chesley S., Ferrari F., Jutzi M., Merrill C.C. (2024). Dimorphos’s Orbit Period Change and Attitude Perturbation due to Its Reshaping after the DART Impact.

[7] Zakharov P.P., Smirnov N.N., Kiselev A.B. (2023). Numerical modelling of high velocity impact problem involving non-linear viscosity. Acta Astronautica.

[8] Liew K.M., Zhao X., Ferreira A.J.M. (2011). A review of meshless methods for laminated and functionally graded plates and shells. Compos. Struct..

[9] Shadloo M.S., Oger G., Le Touzé D. (2016). Smoothed particle hydrodynamics method for fluid flows, towards industrial applications: Motivations, current state, and challenges. Comput. Fluids.

[10] Belytschko T., Krongauz Y., Organ D., Fleming M., Krysl P. (1996). Meshless methods: An overview and recent developments. Comput. Methods Appl. Mech. Engrg.

[11] Buitrago B.L., Santiuste C., Sanchez-Saez S., Barbero E., Navarro C. (2010). Modelling of composite sandwich structures with honeycomb core subjected to high-velocity impact. Compos. Struct..

[12] Ma S., Zhang X., Qiu X.M. (2009). Comparison study of MPM and SPH in modeling hypervelocity impact problems. Int. J. Impact Eng..

[13] Liu M.B., Liu G.R., Zong Z. (2008). An Overview on Smoothed Particle Hydrodynamics. Int. J. Comput. Methods.

[14] Yan X., Zhang Y., Nie J. (2005). Numerical simulation of space debris hypervelocity impact using SPH method. Beijing Hangkong Hangtian Daxue Xuebao/J. Beijing Univ. Aeronaut. Astronaut..

[15] Liu M.B., Liu G.R., Lam K.Y. (2006). Adaptive smoothed particle hydrodynamics for high strain hydrodynamics with material strength. Shock Waves.

[16] Piekutowski A.J., Poormon K.L. (2006). Development of a three-stage, light-gas gun at the University of Dayton Research Institute. Int. J. Impact Eng..

[17] Osher J.E., Barnes G., Chau H.H., Lee R.S., Lee C.H., Speer R., Weingart R.C. (1989). Operating characteristics and modeling of the LLNL 100-kV electric gun. IEEE Trans. Plasma Sci..

[18] LUO B., Zhang X., Long H., Mo J. (2021). Advances on the techniques of ultrahigh-velocity launch above 7 km/s. Explos. Shock Waves.

[19] Violeau D., Rogers B.D. (2016). Smoothed particle hydrodynamics (SPH) for free-surface flows: Past, present and future. J. Hydraul. Res..

[20] Monaghan J.J. (2011). Smoothed Particle Hydrodynamics and Its Diverse Applications. Annu. Rev. Fluid Mech..

[21] Tang J., Sun X. (2017). Simulation Study on Missile Penetration Based on LS—DYNA. IOP Conf. Ser. Mater. Sci. Eng..

[22] Hsueh C.-H., Schmauder S., Chen C.-S., Chawla K.K., Chawla N., Chen W., Kagawa Y. (2020). Handbook of Common Material Parameters for Finite Element Analysis.

[23] Holmquist T.J., Johnson G.R., Cook W.H. A computational constitutive model for concrete subjective to large strain, high strain rates, and high pressure. Proceedings of the 14th International Symposium on Ballistic.

